# Recycling emotions in football: effects of an emotional training program based on psychophysical metaphors with young footballers

**DOI:** 10.3389/fspor.2026.1829114

**Published:** 2026-06-02

**Authors:** P. Rivero-Mena, J. Berastegui-Martínez, A. F. Rodríguez-Hernández

**Affiliations:** 1 Faculty of Psychology, University of La Laguna, Tenerife, Spain; 2 Faculty of Education, Philosophy and Anthropology, HEFA, University of Basque Country, San Sebastian, Spain

**Keywords:** emotional intelligence, emotional training, football training, self-confidence, self-efficacy, youth athletes

## Abstract

**Introduction:**

This study examined the role of emotional intelligence (EI) in self-confidence, self-efficacy, and football-related capacities in youth players, and evaluated the effects of an emotional training program based on psychophysical metaphors.

**Methods:**

A pre-experimental one-group pretest-posttest design was conducted with 70 youth football players. Self-reported and hetero-reported EI, self-confidence, self-efficacy, and football-related capacities were assessed before and after a 4-month emotional training intervention.

**Results:**

EI dimensions showed differential associations with psychological and football variables. Changes associated with the intervention were observed in self-confidence, hetero-reported emotional competencies, and several football-related capacities, with large effect sizes, although self-efficacy did not significantly improve. Given the pre-experimental design, causal attribution cannot be established.

**Discussion:**

Findings highlight the complex and non-linear role of EI in football performance and support the effectiveness of an emotional change-based intervention, offering an innovative alternative to traditional psychological training approaches in developmental football contexts.

## Introduction

In recent years, the scientific community has focused on demonstrating, through empirical studies, how the development of athletes' psychological skills, particularly non-cognitive variables (affective and motivational), directly influences sport performance (1–7). One of the socio-affective variables whose influence on sport performance has been widely examined is self-confidence. La Fratta et al. (8) analyzed the relationship between self-confidence and anxiety, as well as their influence on athletes prior to a football match, using cortisol and oxytocin level measurements. This study demonstrated that lower anxiety levels and higher team self-confidence scores were associated with better performance. The authors concluded that self-confidence is an important factor in sport performance and emphasized that psychological training programs promoting self-confidence may be beneficial for young athletes. Another variable that has been investigated is self-efficacy, understood as the set of beliefs individuals hold regarding their own capabilities to achieve a given outcome (9). Closely related, Vealey (10) developed a conceptual framework of sport confidence, defined as the belief or degree of certainty individuals possess regarding their ability to succeed in sport. Both constructs play an active role in athletes' mental states and may influence performance during training and competition, helping players focus on objectives and sustain beliefs in personal success (11, 12).

A recent meta-analysis based on 31 studies published between 2002 and 2021 concluded that a linear and significant relationship exists between athletes’ confidence levels and sport performance (13). Specifically in women's football, García-Naveira, Ruiz-Barquín, and Ortín (14) highlighted the direct relationship between self-confidence and players' sport performance.

Research consistently indicates that self-confidence increases with age and competitive experience. Studies with large samples of young athletes have shown that older and more experienced competitors exhibit significantly higher confidence levels (15), a pattern also observed in basketball players across different age and competitive categories (16).

### Emotional intelligence and its relationship with sport performance

Emotional intelligence (EI) provides a framework for investigating the influence of emotional experiences on sport performance. One model that conceptualizes EI as an ability is that proposed by Mayer and Salovey (17), which defines emotional intelligence as a set of cognitive abilities for perceiving, using, understanding, and managing emotions effectively. From a complementary perspective, and serving as a reference framework in the present study from a hetero-evaluative standpoint, the model of “competent emotive creativity” (18, 19) conceptualizes EI as a set of intra- and interpersonal competencies that allow individuals to manage emotional experiences effectively and situationally. Given their competence-based nature, these skills are considered trainable. Specifically, these competencies include emotional awareness, emotional change, emotional bonding, and emotional creativity.

A recent study revealed that emotional intelligence influences sport performance through improvements in cognitive processes, identifying EI as a determining factor in performance enhancement (20). Among other aspects, the cited study refers to previous research highlighting that emotional intelligence may contribute to achieving sport success, as athletes with higher EI scores demonstrated better performance. Additionally, EI influences sport performance through strategies used to cope with and manage competition-induced stress (21). The reviewed literature has analyzed various reference variables, including gender. In a study examining the role of age and gender in athletes' emotional intelligence (22), results indicated that while the type of sport practiced did not significantly influence EI among elite athletes, age and gender were relevant variables. Specifically, significant differences favoring women were found in the use of emotions. Similarly, another study analyzing how sport practice affects emotional intelligence found that gender conditioned outcomes, with significant differences in emotional attention and perception, dimensions in which women obtained higher scores (23).

A recent study aimed at analyzing the relationship between emotional intelligence, mental resilience, and mental training in elite athletes considered differences by gender, age, and sport discipline (24). Results showed significant differences in EI levels according to gender, with variations also observed by age and sport branch. Positive associations were identified between mental resilience and EI, as well as between mental training and EI. Specifically, certain groups (e.g., women over 21 years old) exhibited high EI levels, suggesting that gender-age interaction may influence how emotional competencies manifest in competitive performance contexts. Other recent studies also address gender as a relevant variable in emotional intelligence and sport-related capacities. Although some do not directly measure specific physical capacities (e.g., power, strength, speed), they link EI to psychological variables involved in competitive performance (mental resilience, resilience, emotional control), which are considered functional components of overall performance. Additional studies confirm this pattern, showing that gender moderates the relationship between EI and psychological resilience—with men exhibiting higher resilience levels—and that direct gender effects emerge in specific EI dimensions such as emotional well-being, reinforcing the view that emotional competencies are not homogeneous across genders and that particular EI facets may relate differently to performance and adaptation in competitive sport (25, 26).

Multiple studies have focused on analyzing the relationship between emotional intelligence and psychological variables linked to sport performance, such as self-confidence, self-efficacy, and football-specific skills, as well as the correlations among them (27, 28). Specifically, in football, EI plays a fundamental role in self-confidence, self-efficacy, and sport performance. A study conducted with football players found that those with higher EI demonstrated greater emotional resilience under competitive stress, strengthening their confidence when facing sport-related challenges (29). Regarding self-efficacy, this same research indicated that higher self-efficacy linked to specific skills, such as defending or attacking, correlated with better individual performance in those areas. Another study conducted with first-division women football players in Spain observed that “emotional clarity,” an EI dimension, significantly influenced both objective measures (minutes played) and subjective evaluations (coaches' assessments), suggesting that understanding and managing emotions helps optimize both physical and psychological performance on the field (20). Çetin, Adiloğulları, and Şenel (27) conducted research with high-level team sport athletes and provided empirical evidence that emotional intelligence plays a mediating role in the relationship between self-efficacy and self-confidence. Their findings suggest that athletes with higher EI levels more effectively translate their beliefs in personal competence (self-efficacy) into elevated self-confidence levels, offering an explanatory model of how these psychological variables interrelate in team sport contexts. However, the magnitude of the relationship between emotional intelligence and sport performance has been debated. A meta-analysis conducted by Kopp, Jekauc, and Raab (30) concluded that EI shows a positive but small effect size relationship with sport performance. The authors indicated that although EI is consistently associated with performance, its direct influence appears limited and does not depend on the theoretical EI model used. These findings suggest that EI may exert its effects indirectly through intermediate psychological variables such as self-confidence, self-efficacy, or coping processes rather than functioning as a direct predictor of sport performance. In summary, the existing literature suggests that emotional intelligence is significantly related to self-confidence and self-efficacy in athletes and may indirectly influence football-specific capacities through these psychological mechanisms.

### Emotional training in athletes

Within the psychoeducational intervention framework, Berastegui-Martínez and López-Ubis (31) conducted a study with professional women football players to analyze the effects of a socio-emotional competence development program entitled “Aurrera neskak.” The intervention focused on four content areas: emotional awareness, personal autonomy, emotional regulation, and team skills. Results indicated that after the intervention, players perceived positive effects in the expression and perception of their emotions, used coping strategies more frequently, and engaged less frequently in mental withdrawal (a maladaptive strategy) during competitions. These findings confirmed the influence of socio-emotional competencies on coping styles during competition among professional football players and demonstrated that socio-emotional skills can be improved through training, with positive repercussions on performance.

A randomized controlled trial conducted with elite handball athletes showed that a structured intervention based on individual EI profiles produced increases in self-efficacy and competitive anxiety management in the experimental group compared to the control group. This suggests that applying EI-based strategies in counseling contexts has the potential to improve psychological aspects underlying sport effectiveness when aligned with specific performance goals (32).

Additionally, research examining the implementation of emotional training programs in sport contexts found that trait emotional intelligence can be increased through prolonged interventions, although effects may be moderated by characteristics such as age (33). In rugby players, structured EI training was partially effective in increasing EI levels compared to a control group performing video analysis, indicating the feasibility of training emotional competencies in sport and their potential utility in improving psychological processes related to performance.

Other studies have reported a lack of improvement in self-efficacy. Although some show short-term improvements, the literature indicates that transfer to sustained self-efficacy and objective performance depends on training fidelity, number of sessions, contextual practice, type of measures used (self-report vs. objective performance), and participants' baseline levels (30, 34).

Thus, although various psychological interventions and emotional training programs demonstrate positive effects on variables such as emotional regulation, competitive anxiety, and coping, results regarding self-efficacy remain inconsistent. Recent studies suggest that self-efficacy does not automatically improve through emotional training if inhibiting psychological factors—such as competitive anxiety or burnout—are not first reduced, as these factors may mediate the relationship between intervention and self-efficacy (35). Furthermore, the reviewed literature highlights methodological limitations, including heterogeneous measurement instruments, lack of longitudinal follow-up, and contextual differences across sports, which complicate the detection of clear and sustained effects on self-efficacy (36).

In this context, Sulistiyo et al. (37) investigated emotional relationships between coaches and athletes in football and their impact on achievement motivation. They found that coaches' emotional intelligence is crucial in building such relationships, as the coach's ability to understand and respond to athletes' emotional needs can increase athletes' self-efficacy and intrinsic motivation, thereby reinforcing performance. Effective communication was also identified as a key element, enabling coaches to provide constructive feedback and establish open communication channels, creating a climate conducive to the development of achievement motivation. The cited studies also indicate that self-efficacy may depend on sport-specific demands (e.g., return from injury, competitive pressure), and without addressing these stressors, generalized emotional training may not significantly modify it.

Although numerous studies report that emotional intelligence is positively associated with self-confidence and self-efficacy, recent research has indicated that certain EI subdimensions, such as emotional perception (emotional self-awareness), may show negative or weak associations with performance and self-confidence in competitive contexts. According to Zoghlami et al. (38), this inverse relationship may be explained by the possibility that intensely focused emotional awareness may lead to rumination, fear of error, or hypercontrol, which could erode self-confidence and perceived efficacy in high-stress competitive situations. Another possible explanation may involve contextual or task effects, as in sports requiring rapid execution and action confidence, excessive attention to internal sensations may distract or interfere with performance, producing a weak or inverse relationship between emotional self-awareness and self-confidence/self-efficacy. In this regard, research conducted with physical education students found that the emotional perception dimension of EI was negatively correlated with sport performance, whereas other dimensions such as emotional management were positively correlated (38). This implies that high emotional awareness/perception (self-awareness) may be associated with increased focus on internal states that do not facilitate performance or self-confidence, in contrast to emotional management competencies. This pattern aligns with theories such as the self-awareness paradox, which proposes that very high levels of self-awareness may be associated simultaneously with greater introspection and psychological distress, potentially limiting confidence and self-efficacy in demanding situations (39, 40).

Among training methods aimed at acquiring emotional competencies in sport, and specifically in football, mindfulness holds particular relevance (63). The Mindfulness-Based Soccer Program (MBSoccerP), designed for elite athletes and professional football players, demonstrated that mindfulness techniques can be highly beneficial in developing psychological flexibility, reducing anxiety levels, and improving flow states (41). In this line, a recent study (42) showed that football penalty kick takers with higher levels of mindfulness demonstrated greater confidence, lower competitive anxiety, and better attentional control. Self-control has also been shown to be relevant in the relationship between mindfulness and confidence. In an eight-week mindfulness-acceptance-commitment intervention program with elite football players, mindfulness demonstrated a significant indirect effect on improvements in players’ confidence (43).

### The present study

Despite the success reported in the aforementioned studies, the theoretical foundation of these interventions is primarily situated within a cognitive-behavioral framework aimed at modifying individuals' emotional states. To date, however, no training method has emerged in the sport context that is explicitly based on emotional change processes themselves or on alternative strategies more closely related to athletes' emotional expression. Although references within the sport domain are lacking, this alternative approach has demonstrated effectiveness in clinical settings. Greenberg and colleagues argue that Emotion-Focused Therapy (EFT) is grounded in the objective of replacing maladaptive emotions with adaptive ones (44–46).

Rodríguez (18) refers to this affective change process through one of the competencies included in the competent emotive creativity model (19), termed *emotional recycling*. The foundational hypothesis of this innovative competency proposes that, in contrast to the cognitive-behavioral approach, a thought does not change an emotion; at most, it modulates it, and sometimes not even that. On the contrary, emotion often ends up altering thought. From this alternative perspective, we consider that one emotion changes another emotion (18), whether directed toward oneself or shared with another person, provided it is sufficiently powerful to occupy the mental space of the initial emotion. This is what is meant by the concept of emotional recycling. The term is used analogically to explain the competency of modifying one's emotional experiences by using one's own feelings as the raw material for transformation. It refers to the capacity to modify a maladaptive emotional state into a more adaptive one through strategies that compensate for, substitute, or transform it by intentionally activating emotions that possess transformative potential, such as compassion, forgiveness, responsibility, joy, or love (18). This approach to emotional change serves as the reference framework for the present study due to the originality and novelty of the proposal.

To operationalize this perspective, we developed a resource for emotional change termed *psychophysical metaphor* (19). This method represents an adaptation to the sport context of a previous resource known as “life metaphors” (18). It consists of the metaphorical bodily enactment of a socio-affective aspect of the individual, with the aim of promoting emotional change at three levels: (1) awareness of a socio-affective aspect of the individual; (2) substitution of maladaptive emotions with adaptive ones; and (3) emotional creativity, as it involves transcending one's own reality by working with emotional experience from a creative perspective, both in terms of the underlying resource (analogical thinking) and the way emotions are addressed (divergent affectivity). Because it functions as a symbolic parallelism, this approach facilitates access to the implicit domain of emotional functioning, thereby enabling self-awareness. This awareness is further reinforced through bodily representation, which enhances its transformative potential. The objective is for the emotional aspect affecting the individual to be metaphorically enacted within the sport context, and for intervention to occur through psychodramatic representation. For this reason, it is termed a *psychophysical metaphor*, as the content of the metaphor is a specific emotion, and the competency objectives pursued are emotional awareness and emotional change, contextualized within physical and sport activity.

Within this emotional training framework based on emotional change in football players, the present study was developed with the following objectives:

1. To analyze differences in sport self-confidence, sport self-efficacy, emotional intelligence (both self-reported and hetero-reported), and football-specific capacities as a function of gender and age category.
2. To examine the influence of emotional intelligence, from both self-evaluative and hetero-evaluative perspectives, on self-confidence, self-efficacy, and football-specific capacities.
3. To evaluate, through a pre-experimental design, the effect of an emotional training program based on psychophysical metaphors on emotional intelligence, self-confidence, self-efficacy, and football-specific capacities in a sample of football players.

The hypotheses of the present study are as follows:

**Hypothesis 1:** Significant differences exist in sport self-confidence, sport self-efficacy, emotional intelligence (both self-reported and hetero-reported), and football-specific capacities as a function of gender and competitive category (age).
**Hypothesis 2:** Emotional intelligence, from both self-evaluative and hetero-evaluative perspectives, significantly influences self-confidence, self-efficacy, and football-specific capacities.
**Hypothesis 3:** Emotional training based on psychophysical metaphors produces significant improvements in emotional intelligence, self-confidence, self-efficacy, and football-specific capacities.

## Materials and methods

### Study design

A quantitative study with a pre-experimental one-group pretest-posttest design and a correlational component was conducted. This design was selected to explore changes in emotional intelligence following the implementation of an emotional intelligence training program and to examine associations between emotional intelligence and selected psychological and sport-related variables in a youth football context: self-confidence, self-efficacy, and football-related capacities.

### Participants

The sample consisted of 70 youth football players recruited through convenience sampling from a football development program. The mean age was 16.09 years (SD = 2.94). Regarding age-group distribution, the cadet category was more highly represented, comprising 47 participants (57%), compared to the youth/senior group, which included 23 participants (38%). With respect to sex, the sample was relatively balanced, with 33 males (47%) and 37 females (53%), allowing analyses without marked gender bias. The coaches of all participating teams were male, this represents a potential source of systematic bias. All participants were actively training and competing at the time of the study. Inclusion criteria were: (a) being registered as an active football player, (b) regular participation in training sessions and (c) completion of both pretest and posttest assessments. Players who did not complete all assessment phases were excluded from the analyses.

### Measures and instruments

Emotional intelligence (EI) was assessed using the spanish adaptation of the *Trait Meta-Mood Scale,* TMMS-24 (47). The TMMS-24 (48) is a validated self-report questionnaire widely used in adolescent and adult populations. The instrument consists of 24 items distributed across three dimensions of perceived emotional intelligence: emotional attention, emotional clarity, emotional repair (Regulation). Responses are rated on a seven-point Likert-type scale ranging from 1 (not at all true for me) to 7 (totally true for me). In the present study, the TMMS-24 demonstrated adequate psychometric properties and good internal consistency in emotional attention: *α* = .83; emotional clarity: *α* = .82; and emotional repair: *α* = .79.

Emotional competence and emotional creativity were assessed using the *Emotional Competence and Heteroinformed Creativity Questionnaire* (DCREA) (49). The instrument provides an evaluation of emotional and creative competencies based on coaches' observations of players' behavior. The DCREA consists of 8 items rated on a four-point Likert-type scale ranging from 1 (not at all) to 4 (very much). In this study, internal consistency was adequate in emotional awareness: *α* = .84; emotional regulation: *α* = .73; and emotional creativity: *α* = .72.

Self-efficacy was assessed using the *Sport Self-Efficacy Scale (EAD)*, developed by Moritz et al. (50), to evaluate athletes’ perceived ability to successfully cope with sport-specific tasks and demands. The questionnaire consists of 18 items describing situations that may hinder adherence to regular training. Athletes rate each situation according to how capable they feel of maintaining their training routine. Responses are provided on a scale from 0 to 100 (0 = “I cannot maintain it”; 50 = “I can partially maintain it” and 100 = “Totally certain I can maintain it”). An exploratory factor analysis confirmed a unidimensional structure. However, Item 13 was removed due to its negative contribution to internal consistency. The final scale demonstrated adequate reliability (*α* = .90).

Self-confidence was assessed using the *Competition Self-Confidence Questionnaire (CACD)* (51), the spanish adaptation of the Sport Confidence Inventory (SCI) developed by Vealey (10). This instrument evaluates athletes' perceived security in coping with the psychological demands of competition through 7 items assessing thoughts and feelings during competitive situations. Responses are rated on a 7 point Likert scale where 1 = completely disagree and 7 = totally agree. Exploratory factor analysis confirmed a unidimensional structure with adequate reliability (*α* = .85).

An *ad hoc* instrument was developed and validated for the present study to assess *football-related capacities* as evaluated by coaches. The questionnaire examined six capacities: *Physical capacity*, set of qualities underpinning motor performance in football, including strength, power, speed, endurance, and acceleration/change-of-direction ability. These determine effectiveness in high-intensity actions and tolerance to competitive load; *Technical capacity,* efficient and precise mastery of game fundamentals (ball control, passing, dribbling, finishing, shooting), executed economically and under temporal and spatial pressure; *Tactical capacity,* ability to interpret the dynamic game context and make optimal decisions according to offensive and defensive principles; *Emotional capacity*, set of psychological competencies oriented toward self-regulation, including emotional control, self-confidence, resilience, and attentional management; *Relational capacity,* ability to interact effectively with teammates and coaching staff, facilitating communication, cohesion, and collective coordination; *Learning capacity,* disposition and ability to acquire, consolidate, and transfer new technical-tactical knowledge, including openness to feedback and adaptive flexibility.

The questionnaire consisted of 6 items rated on a four-point Likert scale (1 = not at all; 4 = very much). An exploratory factor analysis (EFA) was conducted to examine the underlying structure of the football capacities questionnaire. The results supported a unidimensional structure, with the extracted factor explaining 49.28% of the total variance. Factor loadings ranged from.59 to.85, indicating moderate to high contributions of all indicators to the common construct. The physical capacity dimension was excluded due to its negative impact on the factorial structure. Sampling adequacy was confirmed by a Kaiser-Meyer-Olkin (KMO) index of.76 and a significant Bartlett's test of sphericity [*χ*²(10) = 158.16, *p* < .001]. Internal consistency of the scale was satisfactory (Cronbach's *α* = .82), with no substantial improvement observed after the removal of any item. To validate the factorial structure identified in the EFA, a confirmatory factor analysis (CFA) was conducted using an independent sample. The unidimensional model showed positive and statistically significant factor loadings (*λ* = .26–.50, *p* < .001). Model fit indices indicated an acceptable fit: *χ*²(5) = 13.79, *p* = .017, CFI = .94, TLI = .89, and SRMR = .04. Although the RMSEA value was elevated [RMSEA = .12, 90% CI (.05,.19)], the overall pattern of results supported the factorial validity of the model. The internal consistency of the latent factor was adequate (Cronbach's *α* = .76). Taken together, these findings provide sufficient evidence to support the use of a global football capacities index for subsequent analyses. Despite the psychometric evidence provided, the use of this *ad hoc* instrument represents a limitation of the present study. The elevated RMSEA value (.12) suggests that model fit should be interpreted cautiously, and cross-validation in larger and more heterogeneous samples is needed before broader use of this instrument can be recommended. Its use in the present study was justified by the absence of validated tools specifically designed to assess football-related capacities from a coaching perspective in developmental contexts.

### Emotional intelligence training program (psychophysical metaphors)

The training program was implemented after an initial assessment of participants' socio-affective qualities (emotional, motivational, and personality-related). The intervention was delivered by professionals trained in psychology and/or sport sciences. The program focused on the application of psychophysical metaphors (18), a practice aimed at developing emotional competencies through the body as a mediating element. Targeted components included emotional awareness, emotional regulation, emotional recycling, and the use of emotions to facilitate adaptive behavior during training and competition. The intervention lasted four months and consisted of six sessions per team. Sessions were integrated into the regular training schedule of cadet and youth categories from both professional and non-professional club structures. All teams were coached by male coaches. Sessions followed a three-phase structure: (1) a brief introductory phase (approximately 10 min) in which the targeted emotional competency was introduced conceptually and linked to a specific psychophysical metaphor (e.g., players were invited to identify a maladaptive emotional state they frequently experienced in competition and to represent it bodily through a sport-related movement or posture); (2) a central practical phase (approximately 30–40 min) involving ball work and technical-tactical drills specifically designed to activate the metaphor in context, encouraging players to enact the emotional transformation through physical activity; and (3) a closing reflective phase (approximately 10 min) in which players verbalized their experience, identified the emotional change produced, and discussed how it could be transferred to training and competition situations. The mechanism of change targeted by the intervention was the substitution of maladaptive emotional states with adaptive ones through bodily enactment and metaphorical representation, consistent with the emotional recycling framework (18). In order to provide sufficient detail for replication, the following section describes the structure underlying the elaboration and application of a psychophysical metaphor within a session. The psychophysical metaphor is an adaptation of a previously developed resource known as “life metaphors” (18), here transposed to the sport context. Its functioning operates simultaneously at three levels: at the cognitive level, it constitutes a symbolic analogy that enables understanding of one's emotional reality from a creative-alternative perspective; at the affective level, it facilitates emotional accessibility by circumventing psychological defenses through indirect, symbolically mediated access to implicit emotional functioning; and at the motivational-conative level, it incorporates a playful-communicative dimension, as it operates as a psychophysical game with symbolic meaning that mobilizes the individual and promotes emotional expression. The elaboration and application of a psychophysical metaphor in practice follows a structured sequence of seven steps: **(1) Selection of the target emotion.** The facilitator selects the maladaptive emotion to be addressed through the metaphor (e.g., guilt, fear of failure, frustration after error). **(2) Analogical parallels and symbolic-emotional implications.** Creative analogy is used to generate different types of symbolic parallels and to extract, through guided questioning, implications that specify and concretize the analogical relationship between the emotion and a physical or dramatic image. **(3) Contextualization in a sport execution situation.** The analogical parallels are translated into elements related to a specific physical activity or football context. For example: How could carrying a heavy slab be represented during a training match? Who might embody the role of an internal judge within a football drill, and how would that character behave? **(4) Bodily and/or psychodramatic experimentation.** One of the sport-contextualized analogical parallels is selected and the emotional experience is enacted through physically generated, analogically grounded bodily expression. For example, players complete a small-sided game while physically carrying a teammate on their back—embodying the weight of guilt. (5) **Metaphorical recycling.** Following the enactment of the maladaptive emotion, players bodily represent within the metaphor how that emotion could be substituted or transformed into an adaptive one. Using the same example, instead of bearing a weight, players complete the game holding hands with a partner—enacting collaboration, trust, and shared responsibility in place of guilt. **(6) Creative interpellations.** After both the bodily enactment and the metaphorical recycling, a series of structured reflective questions are applied to deepen the symbolic-emotional experience. These questions address four dimensions: (a) *emotional expression:* What emotions does enacting the metaphor produce? Where in my body did I feel them?; (b) *experiential identification*—How did it feel to embody the assigned role or character?; (c) *symbolic interpretation*—What personal meaning do I assign to the metaphor? What free associations does it generate?; and (d) *vital-sport parallels*—What does this connect to in my everyday life? When have I experienced something similar in training or competition? **(7) Arrival point and sport commitments.** As a conclusion, players are guided to articulate a personal conclusion from the process and to identify specific commitments related to their sport performance—translating the emotional transformation experienced through the metaphor into concrete behavioral intentions for training and competition. This seven-step process was applied flexibly across the six sessions per team, with the specific target emotion and sport context adapted to the characteristics and needs of each group.

### Data collection

Data collection occurred in two phases: pretest and posttest. At baseline, participants completed questionnaires prior to the start of the training program. Upon completion of the intervention, the same instruments were administered under comparable conditions. Questionnaires were completed in group settings under supervision of a research team member to ensure standardized administration. Confidentiality and anonymity were guaranteed. The study was conducted in accordance with the ethical guidelines of the institutional review board and complied with the Declaration of Helsinki. Informed consent was obtained from all participants and, where applicable, from their legal guardians. Participation was voluntary, and participants were informed of their right to withdraw at any time without consequences.

### Statistical analysis

All analyses were conducted using SPSS version 24. Descriptive statistics and reliability coefficients were calculated for all study variables. To examine differences in the study variables according to playing category (juvenile/senior vs. cadet) and gender (male vs. female), Mann–Whitney *U*-tests were conducted due to violations of the normality assumption in several variables. In addition to significance testing, effect sizes were reported using the *r* coefficient (52), interpreted as small (*r* = .10), medium (*r* = .30), and large (*r* ≥ .50), following Cohen's criteria (53). To evaluate the effects of the intervention, a mixed repeated-measures ANOVA was conducted with time (pretest vs. posttest) as the within-subjects factor and gender (male vs. female) as the between-subjects factor. Effect sizes were reported using generalized eta squared (*η²_G_*), following the interpretative benchmarks proposed by Bakeman (54) and Olejnik and Algina (55), whereby values of approximately.02 are considered small, around.13 moderate, and ≥.26 large. Assumptions of sphericity and homogeneity were examined and met prior to conducting the analyses.

## Results

An initial examination of the data is provided in Table 1, which displays the descriptive statistics for the study variables and serves as the basis for the analyses reported below.

**Table 1:** Descriptive statistics of the variables under study.

Variables	Group	*N*	M	SD
Emotional Attention	Youth/Senior	23	24.35	4.88
	Cadets	47	24.83	6.25
	Men	33	23.12	4.68
	Women	37	26.05	6.39
Emotional Clarity	Youth/Senior	23	27.52	4.10
	Cadets	47	26.74	5.94
	Men	33	27.48	5.43
	Women	37	26.57	5.38
Emotional Repair	Youth/Senior	23	29.26	5.40
	Cadets	47	28.19	5.86
	Men	33	27,27	6.83
	Women	37	29.68	4.22
Self-efficacy	Youth/Senior	23	1,314.39	333.67
	Cadets	47	1,324.55	740.01
	Men	33	1,180.48	359.76
	Women	37	1,446.73	786.40
Self-confidence	Youth/Senior	23	46.00	3.00
	Cadets	47	42.06	6.27
	Men	33	44.09	6.62
	Women	37	42.70	4.74
Emotional Awareness	Youth/Senior	23	1.51	0.55
	Cadets	47	1.84	0.63
	Men	33	1.85	0.70
	Women	37	1.62	0.53
Emotional Regulation	Youth/Senior	23	2.26	0.71
	Cadets	47	2.01	0.77
	Men	33	2.44	0.70
	Women	37	1.78	0.66
Emotional Creativity	Youth/Senior	23	1.78	0.74
	Cadets	47	1.84	0.63
	Men	33	1.92	0.72
	Women	37	1.73	0.61
Technical Capacity	Youth/Senior	23	1.83	0.72
	Cadets	47	1.85	0.72
	Men	33	2.09	0.84
	Women	37	1.62	0.49
Tactical Capacity	Youth/Senior	23	1.52	0.73
	Cadets	47	1.60	0.74
	Men	33	1.55	0.87
	Women	37	1.59	0.60
Emotional Capacity	Youth/Senior	23	1.65	0.71
	Cadets	47	1.66	0.81
	Men	33	1.64	0.82
	Women	37	1.68	0.75
Relational Capacity	Youth/Senior	23	2.52	0.79
	Cadets	47	2.36	0.64
	Men	33	2.61	0.75
	Women	37	2.24	0.60
Learning Capacity	Youth/Senior	23	2.57	0.51
	Cadets	47	2.34	0.60
	Men	33	2.64	0.49
	Women	37	2.22	0.58

### Differences in the study variables according to playing competition and players' gender

The analyses revealed significant differences between playing categories in two variables. Youth/senior players showed higher levels of self-confidence (*U* = 342.50, *p* < .05), with a medium effect size (*r* = .37). No significant differences were found between categories in the remaining dimensions of emotional intelligence (TMMS), hetero-reported emotional competence (DCREA), or other football-related capacities.

The analyses revealed significant differences between men and women in several psychological and football-related variables. Women showed higher levels of emotional attention (*U* = 432.50, *p* = .034), with a medium effect size (*r* = .31). In contrast, men obtained higher scores in emotional regulation assessed through hetero-report (*U* = 298, *p* < .001), with a large effect size (*r* = .52). Additionally, male players demonstrated higher levels of relational football competence (*U* = 401, *p* < .05), learning competence (*U* = 356, *p* < .001), and technical competence (*U* = 382, *p* < .01), with medium to large effect sizes (*r* = .32–.45).

### Predictive role of emotional intelligence (self-report)

The player's emotional intelligence (self-report) significantly predicted perceived self-efficacy. The predictive model for self-efficacy was significant, explaining 27% of the variance [*R* = .52, *R^2^* = .27, *F*_(3, 66)_ = 8.01, *p* < .001]. Regarding the individual predictors, emotional attention negatively predicted self-efficacy (*β* = −12.81, *p* = .029), whereas emotional repair emerged as a positive predictor (*β* = 27.41, *p* < .001).

The predictive model for self-confidence was also significant, accounting for 22% of the variance [*R* = .46, *R^2^* = .22, *F*_(3, 66)_ = 6.04, *p* < .001]. In this model, emotional attention was the only significant predictor, showing a negative effect on self-confidence (*β* = −0.23, *p* < .05).

The predictive model for technical football competence was significant, explaining 13% of the variance [*R* = .37, *R^2^* = .13, *F*_(3, 66)_ = 3.39, *p* < .05]. Regarding the individual predictors, emotional attention emerged as a significant predictor and was negatively associated with perceived technical competence (*β* = −0.04, *p* = .01).

The predictive model for emotional football competence showed a trend toward statistical significance, accounting for 11% of the variance [*R* = .33, *R^2^* = .11, *F*_(3, 66)_ = 2.67, *p* < .05]. Within the model, emotional clarity emerged as a significant and positive predictor of emotional football competence (*β* = 0.05, *t* = 2.49, *p* < .05), whereas emotional attention and emotional repair did not show significant effects.

### Predictive role of emotional competence (hetero-report)

The predictive model for self-confidence was significant, accounting for 20% of the variance [*R* = .45, *R^2^* = .20, *F*_(3, 66)_ = 5.53, *p* = .002]. In this model, emotional awareness negatively predicted self-confidence (*β* = −3.25, *p* < .05), whereas emotional creativity positively predicted self-confidence (*β* = 4.14, *p* < .001).

The regression model significantly predicted technical football competence, explaining 18% of the variance [*R* = .42, *R^2^* = .18, *F*_(3, 66)_ = 4.77, *p* = .0005]. Among the predictors, emotional creativity emerged as the only significant predictor and showed a negative association with technical competence (*β* = 0.29, *t* = 2.12, *p* < .05).

The predictive model for tactical football competence was significant, accounting for 29% of the variance [*R* = .54, *R^2^* = .29, *F*_(3, 66)_ = 9.00, *p* < .001]. Emotional awareness (*β* = 0.41, *t* = 2.72, *p* < .01) and emotional creativity (*β* = 0.30, *t* = 2.30, *p* < .05) were significantly and positively associated with tactical competence.

A significant model was found for emotional football competence, explaining 24% of the variance [*R* = .49, *R^2^* = .24, *F*_(3, 66)_ = 7.11, *p* < .001]. Emotional creativity was the only significant predictor, showing a positive relationship with emotional football competence (*β* = 0.30, *t* = 2.11, *p* < .05).

The regression model for relational football competence was also significant, accounting for 31% of the variance [*R* = .55, *R^2^* = .31, *F*_(3, 66)_ = 9.70, *p* < .001]. Two predictors showed significant positive effects: emotional regulation (*β* = 0.25, *t* = 2.32, *p* < .05) and emotional creativity (*β* = 0.27, *t* = 2.23, *p* < .05).

Finally, the predictive model for learning football competence was significant, explaining 32% of the variance [*R* = .57, *R^2^* = .32, *F*_(3, 66)_ = 10.47, *p* < .001]. Both emotional regulation (*β* = 0.24, *t* = 2.68, *p* < .01) and emotional creativity (*β* = 0.25, *t* = 2.56, *p* < .01) were positively associated with this dimension.

### Effects of the intervention on the variables under study

Significant time effects emerged for self-confidence [*F*_(1,40)_ = 122.16, *p* < .001, *η²_G_* = .57], with a large effect size. Scores increased from pre- to posttest in the total sample (*ΔM* = 55.82) [*t*_(40)_ = −11.05, *p* < .001]. No significant gender effect [*F*_(1, 40)_ = 0.05, *p* = .817, *η^2^_G_* = .00] or time × gender interaction [*F*_(1, 40)_ = 0.02, *p* = .889, *η²_G_* = .00] was observed.

For emotional attention, the time effect was not significant [*F*_(1, 40)_ = 3.18, *p* = .082, *η²_G_* = .01]. However, a significant gender effect emerged [*F*_(1,40)_ = 4.47, *p* < .05, *η²_G_* = .09], with a small-to-moderate effect size, indicating higher scores among women, than men with *post hoc* tests confirming this difference [*t*_(40)_ = 2.11, *p* < .05, *ΔM* = 3.31]. The time × gender interaction was not significant [*F*_(1, 40)_ = 2.85, *p* = .099, *η²_G_* = .01].

A significant time effect was found for hetero-report emotional awareness [*F*_(1,40)_ = 886.12, *p* < .001, *η*^2^*_G_* = .85], with a large effect size. *post-hoc* comparisons confirmed the pre-post improvement [*t*_(40)_ = 2.89; *p* < .01; *ΔM* = 4.30]. In addition, a significant gender effect was observed [*F*_(1,40)_ = 8.34, *p* *<* .01, *η*^2^*_G_* = .13], with men scoring higher than women [*t*_(40)_ = 2.89, *p* < .05, *ΔM* = 0.71]. The time × gender interaction was not significant [*F*_(1, 40)_ = 1.75, *p* = .193, *η*^2^*_G_* = .01].

Similarly, hetero-report emotional regulation showed a significant time effect [*F*_(1,40)_ = 177.73, *p* < .001, *η*^2^*_G_* = .57], with a large effect size, and a pre-post increase [*t*_(40)_ = 13.33; *p* < .001; *ΔM* = 2.10]. The gender effect was also significant [*F*_(1,40)_ = 12.79, *p* *<* *.*001, *η*^2^*_G_* = .18], again indicating higher scores in men [*t*_(40)_ = 3.58, *p* < .01, *ΔM* = 0.87]. The time × gender interaction was not significant [*F*_(1, 40)_ = 1.55, *p* = .221, *η*^2^*_G_* = .01].

Hetero-report Emotional creativity displayed a significant time effect [*F*_(1,40)_ = 607.48, *p* < .001, *η*^2^*_G_* = .80], with a large effect size and significant pre-post gain [*t*_(40)_ = 24.65, *p* < .001, *ΔM* = 4.55]. Neither the gender effect [*F*_(1, 40)_ = 2.58, *p* = .116, *η*^2^*_G_* = .05], nor the time × gender interaction [*F*_(1, 40)_ = 1.16, *p* = .289, *η*^2^*_G_* = .01], reached significance.

Regarding football-related capacities, technical condition showed a significant time effect [*F*_(1,40)_ = 11.73, *p* < .001, *η*^2^*_G_* = .03], with a small effect size, and a time × gender interaction [*F*_(1,40)_ = 8.88, *p* < .01, *η*^2^*_G_* = .03], with a small effect size. Follow-up comparisons indicated that women improved from pre- to posttest [*t*_(40)_ = 3.99; *p* < .001, *ΔM* = 0.54], whereas men exhibited minimal change (*ΔM* = 0.04). The gender main effect was not significant [*F*_(1, 40)_ = 2.13, *p* = .152, *η*^2^*_G_* = .04].

A significant time effect was also found for tactical capacity [*F*_(1,40)_ = 23.10, *p* < .001, *η*^2^*_G_* = .10], with a moderate effect size, indicating higher posttest scores. Both men [*t*_(40)_ = 3.96; *p* < .001, *ΔM* = 0.52] and women [*t*_(40)_ = 3.04; *p* < .01, *ΔM* = 0.53] improved their posttest scores compared to the pretest. No significant gender effect [*F*_(1,40)_ = 0.38, *p* = .543, *η*^2^*_G_* = .01] or interaction [*F*_(1, 40)_ = 0.00, *p* = .946, *η*^2^*_G_* = .00] was observed.

Football-related emotional capacity improved over time [*F*_(1,40)_ = 12.35, *p* < .001, *η*^2^*_G_* = .08], with a small-to-moderate effect size, increasing from pre- to posttest [*t*_(40)_ = 3.51; *p* < .001, *ΔM* = 0.42]. No significant gender effect [*F*_(1,40)_ = 0.01, *p* = .938, *η*^2^*_G_* = .00] or interaction was found [*F*_(1, 40)_ = 0.03, *p* = .854, *η*^2^*_G_* = .00].

For learning capacity, the time effect was not significant [*F*_(1,40)_ = 0.14, *p* = .709, *η*^2^*_G_* = .00]; however, a time × gender interaction emerged [*F*_(1,40)_ = 8.49, *p* < .01, *η*^2^*_G_* = .04], with a small effect size. *post hoc* tests indicated that men showed a significant reduction from pre- to posttest [*t*_(40)_ = –2.75, *p* < .05, *ΔM* = − 0.26], whereas women showed a modest, non-significant increase *[t*_(40)_ = 1.58, *p* = .400*, ΔM* = 0.20]. The gender main effect was not significant [*F*_(1,40)_ = 1.16, *p* = .*289*, *η*^2^*_G_* = .02].

Finally, across the remaining variables — emotional clarity, emotional repair, self-efficacy and relational capacity — no significant time effects, gender effects, or time × gender interactions were observed. It should be noted that, given the pre-experimental nature of the design, the observed changes cannot be attributed exclusively to the intervention, as alternative explanations such as maturation, competitive exposure, or regular training effects cannot be ruled out.

Table 2 reports the mixed repeated-measures ANOVA results, including the effects of time, gender, and their interaction on all psychological and football-related variables, as well as corresponding effect sizes and *post-hoc* comparisons.

**Table 2:** Mixed repeated-measures ANOVA examining time (Pre-post) × gender effects.

Variable	Time Effect F_(1,40)_	*p*	*η*²G	Gender Effect F_(1,40)_	*p*	η²G	Time × Gender F_(1,40_)	*p*	η²G	Post hoc/Interpretation
Self-confidence	122.16	<.001	.57	0.05	.81	.00	0.02	.88	.00	Pre-post *ΔM* = 55.82; *t_(_*_40)_ = −11.05, *p* < .001
Emotional attention	3.18	.08	.01	4.47	<.05	.09	2.85	.09	.01	Women > Men: *ΔM* = 3.31; *t*_(40)_ = 2.11, *p* < .05
Emotional awareness	886.12	<.001	.85	8.34	<.01	.13	1.75	.19	.01	Pre-post *ΔM* = 4.30; *t*(40) = 2.89, *p* < .01; Men > Women *ΔM* = 0.71; *t*_(40)_ = 2.89, *p* < .05
Emotional regulation	177.73	<.001	.57	12.79	<.001	.18	1.55	.22	.01	Pre-post *ΔM* = 2.10; *t*_(40)_ = 13.33, *p* < .001; Men > Women *ΔM* = 0.87; *t*_(40)_ = 3.58, *p* < .01
Emotional creativity	607.48	<.001	.80	2.58	.11	.05	1.16	.28	.01	Pre-post *ΔM* = 4.55; *t*_(40)_ = 24.65, *p* < .001
Technical capacity	11.73	<.001	.03	2.13	.15	.04	8.88	<.01	.03	Interaction: Women *ΔM* = 0.54; *t*_(40)_ = 3.99, *p* < .001; Men *ΔM* = 0.04
Tactical capacity	23.10	<.001	.10	0.38	.54	.01	0.00	.94	.00	Pre-post Men *ΔM* = 0.52; *t*_(40_) = 3.96, *p* < .001; Women *ΔM* = 0.53; t_(40)_ = 3.04, *p* < .01
Emotional capacity	12.35	<.001	.08	0.01	.93	.00	0.03	.85	.00	Pre-post *ΔM* = 0.42; *t*_(40)_ = 3.51, *p* < .001
Learning capacity	0.14	.70	.00	1.16	.28	.02	8.49	<.01	.04	Interaction: Men *ΔM* = −0.26; *t*_(40)_ = −2.75, *p* < .05; Women *ΔM* = 0.20; *t*_(40)_ = 1.58, *p* = .40
Emotional clarity	0.32	.57	.00	0.10	.75	.00	0.23	.63	.00	No significant effects
Emotional repair	0.54	.46	.00	2.03	.16	.01	1.68	.20	.03	No significant effects
Self-efficacy	0.04	.83	.00	0.02	.90	.00	3.57	.06	.07	No significant effects
Relational capacity	0.33	.56	.00	1.05	0.31	.00	1.83	.18	.04	No significant effects

## Discussion

The present study pursued three primary objectives: (1) to analyze differences in self-confidence, self-efficacy, emotional intelligence (EI) -both self-reported and hetero-reported—and football-related capacities as a function of gender and competitive category; (2) to examine the influence of EI on self-confidence, self-efficacy, and football-related capacities; and (3) to evaluate the effects of an emotional training program based on psychophysical metaphors on these variables. Overall, the findings indicate that: (a) according to competitive category and gender, significant differences exist in self-confidence and in specific dimensions of emotional intelligence and football-related capacities; (b) different EI dimensions play a differential role within the football context, particularly in developmental stages; and (c) these competencies are susceptible to improvement through systematic training methods. Taken together, these findings are consistent with previous literature linking emotional variables to sport performance (2, 3, 13, 20) and provide novel evidence regarding the effectiveness of an approach centered on emotional change.

Regarding the first objective, results showed that youth/senior players displayed higher levels of self-confidence prior to the intervention compared to cadet players. This finding aligns with previous research indicating a progressive increase in sport confidence as competitive experience and exposure to successful performance situations accumulate (15, 16). From a sport development perspective, greater exposure to demanding competitive contexts may facilitate processes of internal validation and perceived competence, thereby reinforcing self-confidence. Repeated competitive experiences, exposure to real performance pressure, and the gradual assumption of greater responsibility roles contribute to the internalization of success expectations and perceived task control. In contrast, in early developmental categories, self-confidence tends to be more fragile and dependent on external feedback, rendering it more vulnerable to competitive uncertainty. It should be noted, however, that the relatively small size of the youth/senior subsample (*n* = 23) reduces statistical power for subgroup analyses and limits the conclusions that can be drawn regarding age-related differences. Furthermore, age variability within categories could not be fully examined given the available data. Future studies with larger and more age-stratified samples would allow more granular developmental analyses of self-confidence and emotional competencies across competitive stages.

With respect to gender, a differential pattern emerged across EI dimensions: women obtained higher scores in emotional attention, whereas men showed higher levels of hetero-reported emotional regulation. These findings are consistent with prior studies describing women's greater tendency toward emotional perception and monitoring (22, 23), in contrast to men's stronger orientation toward functional emotional regulation in performance contexts (24, 26).

Regarding football-related capacities, men obtained higher scores in technical, relational, and learning capacities. Similar findings have been interpreted in the literature as reflecting structural inequalities in access to training opportunities, competitive continuity, and performance expectations (14, 25).

From our perspective, these findings may be partially associated with differences in emotional socialization and perceived demands within the sport context, as well as structural inequalities in access to developmental opportunities, training volume, and competitive continuity in football—particularly in mixed or developmental settings. This interpretation is linked to the methodological characteristics of the assessment: the tendency of women to report greater emotional attention was identified through a self-report instrument, meaning that women perceive themselves as placing greater emphasis on emotional monitoring. In contrast, the higher levels of emotional regulation, relational competence, learning capacity, and technical aptitude observed in men were derived from coaches' evaluations, all of whom were male. The fact that these capacities were assessed exclusively by male coaches introduces a potential source of systematic bias that must be carefully considered when interpreting the results, particularly in gender comparisons. Research has documented that male coaches tend to subtly communicate discriminatory views about female athletes' capabilities, making them feel less competent compared to their male counterparts (56). In line with these findings, qualitative research has shown that male coaches operating in mixed-gender sport contexts tend to interpret female athletes' emotional expressiveness as a performance liability, applying evaluative frameworks grounded in masculine norms of competitive behavior (57). Taken together, these findings suggest that the higher scores observed in male players in technical, relational, and learning capacities—all evaluated by male coaches—may partially reflect evaluative tendencies rooted in shared gender socialization and differential performance expectations, rather than actual differences in competence. Future studies should incorporate gender-diverse evaluation teams to minimize this source of systematic bias.

Overall, these findings partially confirm the initial hypothesis, as significant differences were observed in self-confidence, emotional intelligence, and football-related capacities according to category and gender; however, not all variables followed the expected pattern or direction. From a practical standpoint, these results highlight the need to design psychological interventions that are sensitive to developmental stage and gender, providing relevant evidence for the personalization of psychological training in football.

One of the most relevant findings related to the second objective concerns the differential effects of EI dimensions. From the self-report perspective, emotional attention showed negative associations with self-confidence, self-efficacy, and technical capacity, whereas emotional repair was positively associated with self-efficacy. In hetero-reported models, this differential effect was observed only for self-confidence: emotional awareness was negatively associated, whereas emotional creativity was positively associated.

These findings support the notion that heightened emotional awareness, when not accompanied by effective regulatory strategies, may be associated with rumination, doubt, or excessive emotional analysis, thereby interfering with perceived competence and personal control. Recent studies suggest that emotional perception may function as a risk factor in high-stress competitive contexts when not integrated within a functional emotional profile (38). High levels of emotional self-awareness may promote excessive focus on internal states that do not facilitate performance or confidence—a pattern consistent with the self-awareness paradox (39, 40), discussed in greater detail below in relation to hetero-reported findings. Conversely, dimensions linked to adaptive regulation and emotional creativity appear to facilitate flexible interpretation of competitive demands, promoting confidence and perceived personal efficacy. This pattern aligns with previous mediational models positioning emotional intelligence as a moderator of the impact of self-efficacy on self-confidence (27).

Regarding the influence of EI on football-related capacities, results also indicate significant associations, though again not uniformly. From the self-report perspective, emotional attention was negatively associated with technical capacity, whereas emotional clarity positively predicted emotional capacity. Particularly noteworthy, from the hetero-reported perspective, is the generalized positive predictive role of emotional creativity across all football-related capacities.

The positive influence of emotional creativity is consistent with models emphasizing psychological flexibility, resilience, and adaptive coping in sport performance (21, 25). This finding is especially relevant in football, a sport characterized by uncertainty and rapid decision-making. At a mechanistic level, emotional creativity is proposed to facilitate adaptive responses in dynamic game situations by enabling players to reframe competitive demands flexibly rather than reacting with fixed emotional patterns. Emotional regulation, which positively predicted relational and learning capacities, is linked to the ability to maintain effective interpersonal functioning and openness to feedback even under competitive stress, consistent with models emphasizing its role in team cohesion and coachability (21). Conversely, emotional awareness showed a negative association with self-confidence in the hetero-reported model, which can be understood through the self-awareness paradox (39, 40): heightened awareness of emotional states, when not integrated within a functional regulatory profile, may increase rumination and interfere with confident and automatic performance execution. Taken together, these findings suggest that the improvements in hetero-reported emotional competencies observed following the intervention may have contributed to the parallel improvements in football-related capacities through these mechanisms, while acknowledging that the pre-experimental design does not permit causal conclusions. Therefore, the second hypothesis is partially confirmed. Emotional intelligence demonstrated a significant influence on self-confidence and football-related capacities; however, some dimensions -such as emotional attention- exerted effects opposite to those expected.

Overall, these findings indicate that EI influences self-confidence, self-efficacy, and football capacities in a significant but non-homogeneous manner. From an applied perspective, the results suggest that not all emotional dimensions are functionally beneficial for performance, and that elevated emotional attention without adequate regulation may constitute a psychological risk factor. These findings orient intervention efforts toward the development of functional emotional competencies, particularly regulation and emotional creativity, providing empirical support for optimizing football performance.

Finally, regarding the third objective, analyses showed significant changes associated with the intervention in self-confidence and hetero-reported emotional competencies (awareness, regulation, and emotional creativity), with large effect sizes. However, given the absence of a control group, these results should be interpreted with caution, as the observed improvements may be partially attributable to factors independent of the intervention, such as maturation or regular training exposure. These results are consistent with studies demonstrating the trainability of emotional intelligence in sport contexts (31–33). No significant gender interactions were observed for these variables, although gender as an independent factor differentiated emotional attention, awareness, and regulation, as previously noted.

Concerning football-related capacities, significant improvements were observed in technical, tactical, and emotional capacities, with a particularly notable effect in women's technical capacity. No significant changes were found in relational capacity, and learning capacity showed a Time × Gender interaction characterized by improvement in women and a decrease in men. In line with these findings, previous research suggests that emotional programs may have a differential impact depending on contextual and participant characteristics (58–60).

Although the emotional training program demonstrated positive effects on most dependent variables, no significant improvements were observed in self-efficacy. This result is consistent with literature highlighting the complexity of self-efficacy as a construct (30, 34, 35) and its dependence on mediating factors such as training fidelity, number of sessions, contextual practice, type of measurement, and participants' baseline levels. Several complementary explanations may account for this absence of change. First, self-efficacy as conceptualized by Bandura (9) is primarily shaped by mastery experiences, vicarious learning, verbal persuasion, and physiological states—sources that a short-term emotion-focused intervention may not sufficiently activate. Second, the self-report nature of the measure (EAD) may limit its sensitivity to change in the absence of direct objective performance feedback. Third, the differential pattern observed—whereby self-confidence improved significantly while self-efficacy did not—is theoretically meaningful: although related, these constructs tap into distinct psychological processes, with self-confidence reflecting a more global sense of security and self-efficacy referring to task-specific competence beliefs. Their differential responsiveness to emotional training represents a finding worthy of further investigation. Finally, consistent with Tang et al. (35), self-efficacy may not improve through emotional training if inhibiting factors such as competitive anxiety or burnout are not first addressed, as these may mediate the relationship between the intervention and perceived efficacy. A more specific theoretical explanation for this absence of change can be found in the structural mismatch between the mechanism of change underlying psychophysical metaphors and the sources through which self-efficacy is built. According to Bandura's social cognitive theory (61), self-efficacy beliefs are shaped by four hierarchically ordered sources: mastery experiences, vicarious experiences, verbal persuasion, and physiological and emotional states—with mastery experience constituting the most influential source, followed in descending order by the remaining three (61). Psychophysical metaphors operate primarily through emotional transformation and bodily enactment, targeting emotional awareness and the substitution of maladaptive emotional states—mechanisms that correspond most directly to the fourth and least influential source in Bandura's hierarchy. While the reflective phase of each session may have provided some degree of verbal persuasion, the intervention did not systematically generate task-specific mastery experiences or structured vicarious learning opportunities directly linked to perceived football competence. This is consistent with Bandura's own position that the most effective psychological procedure for building self-efficacy is performance-based (61), that is, grounded in direct experiences of successful task execution. Furthermore, an improvement in emotional regulation does not automatically translate into an increase in self-efficacy unless the player cognitively reinterprets that emotional change as evidence of personal competence in specific sport situations (61). The intervention may have enhanced the players' capacity to manage their emotional states, but without explicit feedback linking that improvement to perceived task mastery, the pathway to self-efficacy change may not have been sufficiently activated. This interpretation suggests that future interventions aiming to simultaneously improve emotional competencies and self-efficacy should explicitly incorporate performance-based components—such as structured mastery tasks with progressive difficulty, guided self-monitoring of improvement, and systematic use of vicarious modeling—alongside emotion-focused strategies. Accordingly, the third hypothesis is partially confirmed. Changes consistent with the intervention were observed in self-confidence, emotional intelligence, and football-related capacities, but not in self-efficacy. These findings should be interpreted within the constraints of the pre-experimental design, which does not permit causal attribution. These findings align with recent studies demonstrating the trainability of emotional competencies and their positive impact on psychological variables relevant to sport performance (31, 33, 62). Moreover, unlike previous studies reporting small effect sizes (30), the magnitude of the effects observed in the present research was notably large. These large effect sizes, however, should be interpreted with caution in the context of a pre-experimental design. In particular, hetero-reported measures may be more sensitive to change than self-reported ones, as coaches who observed the sessions may have been more attuned to behavioral shifts, potentially contributing to inflated effect sizes. Future controlled studies are needed to confirm whether such magnitudes are replicated under more rigorous experimental conditions. It is also important to note that, except for self-confidence, the variables that improved were evaluated from the coaches’ perspective, which adds a relative degree of external validation to the findings. Taken together, these results and their conditions may challenge the hypothesis that emotional intelligence does not exert a direct influence on sport performance and instead operates only through mediating variables, independently of the theoretical model used to design the intervention. The data obtained may be interpreted—as a hypothesis to be tested in future studies with more robust experimental designs—as suggesting that the observed changes are consistent with the competency-based evaluation approach and the emotional change orientation of the intervention, particularly through the application of psychophysical metaphors. However, causal claims cannot be made on the basis of the current design.

## Conclusion

Overall, the results confirm that emotional intelligence plays a relevant, albeit complex and non-linear, role in self-confidence, self-efficacy, and football-related capacities. Importantly, the findings highlight that not all dimensions of emotional intelligence are functionally beneficial in the same way. Systematic intervention from an emotional change perspective—through the application of psychophysical metaphors—was associated with improvements in self-confidence, emotional competencies, and football-related capacities, though causal inference is limited by the pre-experimental nature of the design. These findings have direct implications for the design of emotional intervention programs in developmental football contexts. Specifically, they offer an innovative alternative to traditional cognitive-behavioral approaches and open new avenues for application and research in sport psychology. By emphasizing emotional transformation rather than solely cognitive restructuring, this approach contributes to a more comprehensive understanding of psychological training in football and expands the methodological repertoire available to practitioners.

### Study limitations and future directions

The present work should be considered a preliminary approach to addressing the research problem, and its findings must be interpreted in light of several methodological limitations. The most substantial limitation concerns the pre-experimental one-group pretest-posttest design. The absence of a control group prevents direct causal attribution of the observed changes to the intervention, as alternative explanations—including natural maturation, regular training exposure, and competitive experience accumulated over the four-month period—cannot be ruled out. Although the pre-experimental design is common in exploratory intervention research and was appropriate given the applied and developmental context of the study, it represents a fundamental constraint on internal validity. Future studies should incorporate randomized controlled trial designs with matched control groups to allow direct comparison between intervention and non-intervention conditions and to strengthen causal inference. Longitudinal follow-up assessments would also be valuable to examine the stability of the observed changes over time, an aspect that the present design does not address. A second limitation concerns the use of an *ad hoc* instrument to assess football-related capacities. This instrument was developed in response to the absence of validated tools specifically designed to evaluate these capacities from a coaching perspective in developmental football contexts. Although the psychometric evaluation yielded promising preliminary results—including a unidimensional factor structure, satisfactory internal consistency, and acceptable confirmatory fit indices—the elevated RMSEA value (.12) indicates that the model fit should be interpreted with caution. The instrument's construct validity remains limited, and cross-validation in larger, more diverse, and more heterogeneous samples is needed before broader use can be recommended. Furthermore, as all evaluations were conducted exclusively by male coaches, the possibility of systematic gender bias in the assessment of football-related capacities cannot be excluded. Future studies should incorporate gender-diverse evaluation teams and, ideally, validated observational instruments with established inter-rater reliability. A third limitation relates to the sample size and its implications for subgroup analyses. Although the total sample of 70 participants is adequate for an exploratory study of this nature, the subdivision by age category results in a relatively small youth/senior subsample (*n* = 23), which reduces statistical power for age-related comparisons and limits the conclusions that can be drawn regarding developmental differences. Similarly, while the gender distribution was relatively balanced at the aggregate level, the interaction between age category and gender produced subgroups whose sizes were insufficient for fully powered analyses. This means that certain potentially meaningful differences—for example, whether the effect of the intervention varies across age and gender simultaneously—could not be examined with adequate precision. These constraints should be explicitly acknowledged when interpreting the findings related to Hypothesis 1, and future studies should employ larger and more age-stratified samples to allow more granular analyses of developmental and gender-related patterns in emotional competencies and football-related capacities.Finally, the study was conducted within a single cultural and organizational context—a football development program in Spain—which limits the generalizability of the findings to other populations, sport systems, and cultural settings. The sample was also drawn through convenience sampling, which introduces potential selection bias. Future research should seek to replicate these findings in more diverse samples across different competitive levels, age groups, and cultural contexts. Notwithstanding these limitations, the present study offers novel evidence supporting the relevance of incorporating an emotional change-based approach into football training. Beyond its contribution to optimizing sport performance, this framework may also function as a preventive strategy against potential mental health difficulties, which is particularly relevant given the high-pressure environment, elevated performance demands, and frequent lack of affective support and psychological guidance that characterize many sport contexts. From this perspective, integrating psychophysical metaphors and emotional recycling methodologies into football training may contribute not only to performance excellence but also to the psychological well-being and long-term development of athletes.

## Data Availability

The raw data supporting the conclusions of this article will be made available by the authors, without undue reservation.
